# Epidemiology, biometric features and risk factors of pseudoexfoliation syndrome among cataract patients: a hospital-based study in Apulia

**DOI:** 10.1007/s00417-026-07116-4

**Published:** 2026-01-30

**Authors:** Flavio Cassano, Gianluca Besozzi, Tiziana Rizzo, Michele Maffia, Maria Carmela Costa

**Affiliations:** 1 Univerisità del Salento, Via Lecce-Monteroni, Monteroni di Lecce, LE 73047 Italy; 2 P.O. Vito Fazzi, U.O.C. Oftalmologia, Piazza Filippo Muratore, Lecce, Italy

**Keywords:** Pseudoexfoliation syndrome, Cataract, Epidemiology, Risk factors, Neutrophil-to-lymphocyte ratio, Biometry

## Abstract

**Purpose:**

to contextualize the prevalence of pseudoexfoliation syndrome (PEX) through a systematic literature review and to evaluate its epidemiology, ocular biometric features, and potential systemic associations in a hospital-based cohort of cataract patients from Southern Italy, with particular focus on the neutrophil-to-lymphocyte ratio (NLR) as a marker of systemic inflammation and surgical risk.

**Methods:**

this study consisted of two components. First, a systematic literature review was conducted according to PRISMA principles to summarize reported PEX prevalence across different populations and geographic regions. Second, a retrospective cross-sectional cohort study was performed including consecutive cataract patients evaluated at a tertiary referral center in Apulia (Italy) between January 2024 and September 2025. Demographic data, ocular biometry, corneal parameters, systemic comorbidities, and preoperative NLR values were collected. Eye-level outcomes were analyzed using linear mixed-effects models with patient-level random intercepts, adjusting for age, sex, and eye side. Associations between NLR, biometric parameters, and surgical complications were assessed using correlation analyses and non-parametric tests.In the absence of a pre-specified primary outcome, ACD was selected a posteriori as the primary biometric endpoint.

**Results:**

the literature review confirmed marked heterogeneity in reported PEX prevalence worldwide, ranging from < 1% to > 30%, largely influenced by study design and population characteristics. In the cohort study, 99 of 3,736 cataract patients were diagnosed with PEX, yielding a prevalence of 2.65%. PEX patients were significantly older than non-PEX patients, with no sex predominance. After adjustment, PEX was not independently associated with axial length, anterior chamber depth, aqueous depth, lens thickness, central corneal thickness or endothelial cell density. NLR showed no significant correlation with ocular biometric parameters and did not differ between patients with and without intraoperative or postoperative complications.

**Conclusion:**

PEX was relatively uncommon in this Southern Italian cohort and primarily age-related. No association was found between PEX and standard biometric features or between systemic inflammation (NLR) and surgical risk. Larger, multicenter studies integrating genetic and inflammatory markers are warranted to further elucidate PEX pathogenesis.

## Key messages

**What is known:**

- Pseudoexfoliation (PEX) is prevalent in older adults and increases intraoperative difficulty during cataract surgery; reported prevalence varies widely across regions and study designs.
- The neutrophil-to-lymphocyte ratio (NLR) has been proposed as an inflammatory biomarker potentially linked to surgical risk in PEX.

**What is new:**

- In this hospital-based cohort from Apulia, PEX showed a low prevalence (2.65%) among cataract surgery patients.
- Using linear mixed-effects models (patient-level random intercept) adjusted for age, sex, and eye side, PEX was not independently associated with ACD (primary outcome) and other standard biometric parameters (AL, AQD, LT, CCT).
- NLR was not associated with ocular biometry or with the occurrence of intra- or postoperative complications.

## Introduction

Pseudoexfoliation syndrome (PEX) is a systemic disorder affecting elastin formation. It leads to the abnormal deposition of small, gray-white fibrillar aggregates (resembling amyloid) on the lens capsule, iris, and other anterior segment structures. These deposits can also manifest systemically, potentially involving the skin, heart, and lungs.

The distinctive PEX fibrils are produced at multiple sites by various intraocular cell types, including the pre-equatorial lens epithelium, non-pigmented ciliary epithelium, trabecular and corneal endothelium, vascular endothelial cells, and nearly all cell types within the iris [1].

Intraocular manifestations include [1] phakopathy, with phakodonesis and (sub)luxation, cyclopathy/zonulopathy, iridopathy, that may manifest as vasculopathy, with features as defects in the blood-aqueous barrier, anterior chamber hypoxia, iris rigidity, poor mydriasis, posterior synechiae, and pigment dispersion; trabeculopathy, that may generate glaucoma, and keratopathy, with reduced endothelial cell count, endothelial decompensation, corneal endothelial proliferation over the trabecular meshwork. PEX syndrome is the leading identifiable cause of glaucoma, contributing significantly to both open-angle and angle-closure glaucoma in many populations. Iridolenticular friction and disruption of the iris pigment epithelium result in pigment and exfoliative material deposition in the trabecular meshwork, exacerbating pressure elevation through degenerative changes in Schlemm’s canal and adjacent tissues. Narrow angles and angle-closure are common, often linked to pupillary block caused by posterior synechiae, iris rigidity, anterior lens displacement due to zonular instability, or cataract-related lens enlargement [2].

The reported prevalence of PEX syndrome varies widely, even within different regions of the same country, due to factors such as patient selection, the ethnic composition of the population, the diagnostic criteria used, and the thoroughness of clinical examinations [3].

PEX has been shown to increase with age [4]. Other purposed risk factors are solar radiation [5], vascular pathologies [6] and HLA [7]. Recently, a novel biomarker for PEX has been found in the Neutrophil/Lymphocyte ratio (NLR) [8, 9].

Systemic inflammation, as indicated by an elevated NLR, may further exacerbate these risks by contributing to impaired wound healing, heightened inflammatory responses, or increased susceptibility to postoperative complications like cystoid macular edema or endophthalmitis. Moreover, an intense state of inflammation could determine a higher risk of perioperative complications [10].

Preoperative PEX diagnosis is critical to avoid several intraoperative complications, such as poor pupil dilation, posterior capsule rupture and vitreous loss [11].

The present work consists of two complementary components:

1. **A systematic literature review**, conducted according to PRISMA principles, summarizing the prevalence of PEX reported in adult populations worldwide and characterizing demographic variability across geographic regions.
2. **A hospital-based cohort study** of consecutive cataract patients from a Southern Italian tertiary center, designed and reported in accordance with STROBE recommendations.

This study aimed to:

- determine the prevalence and demographic profile of PEX in our catchment area,
- examine ocular biometric features associated with PEX using mixed-effects modeling,
- evaluate the relationship between systemic inflammation (NLR) and ocular parameters
- assess whether NLR is associated with intraoperative or postoperative complications among PEX patients.

By integrating findings from the systematic review with detailed cohort-level data, this project provides a coherent narrative describing both the epidemiological context and the local clinical profile of PEX in an area of Southern Italy where such data have not previously been reported.

## Materials and methods

### Study design overview

This project included two complementary components:

1. **A systematic literature review**, conducted according to PRISMA 2020 principles.
2. **A retrospective hospital-based cohort study**, conducted and reported according to STROBE guidelines.

This dual structure enabled both contextualization of PEX prevalence worldwide and detailed characterization of PEX patients undergoing cataract surgery in our center.

### Literature review procedure

The literature review followed the principles of the PRISMA 2020 statement for transparent reporting of systematic searches.

Electronic searches were conducted in PubMed, Scopus, and Google Scholar, using the following keywords and Boolean combinations:

(“pseudoexfoliation” OR “PEX”) AND (“prevalence” OR “epidemiology”) AND (“cataract” OR “ocular”).

The last search was performed on May 31 st 2025.

Two reviewers (F.C. and G.B.) independently screened all titles and abstracts, and disagreements were resolved by consensus. Reference lists of included papers were manually reviewed to identify additional relevant studies.

#### Eligibility criteria

Inclusion:

- Observational (population-based or hospital-based) studies.
- Reported PEX prevalence.
- English language.

Exclusion:

- Case reports, narrative reviews.
- Non-ocular populations.
- Insufficient prevalence data.
- Non-English articles.

#### Study selection

Two independent reviewers screened titles and abstracts; disagreements were resolved by consensus. Reference lists were manually reviewed to identify additional studies.

#### Data extraction

From each included study we collected:

- sample size.
- mean age.
- sex distribution.
- type of population (clinic-based, cataract group, general population).
- PEX prevalence.
- associated factors.

This review was not pre-registered. Risk of publication bias and study quality were not formally assessed, as the purpose was descriptive rather than quantitative synthesis.

### Hospital-based cohort study (STROBE Framework)

#### Setting and participants

This retrospective cross-sectional study included all consecutive patients evaluated for cataract surgery at the Ophthalmology Unit of “Vito Fazzi” Hospital (Lecce, Italy) between January 2024 and September 2025.

#### Inclusion criteria

- Patients scheduled for cataract surgery during the study period.
- Phakic eyes at baseline.
- Availability of complete preoperative and postoperative records.

#### Exclusion criteria

- Missing or incomplete medical documentation.
- Ocular comorbidities influencing surgical outcomes (advanced glaucoma, uveitis, retinal detachment).
- Combined surgical procedures (e.g., cataract + glaucoma surgery).

#### Data collection

Collected variables included:

- Demographics: age, sex.
- Ocular biometry: ACD, AL, AQD, LT (IOLMASTER 700, Zeiss, Oberkochen, Germany).
- Corneal parameters: ECD, CCT (Perseus, CSO, Scandicci, Italy).
- Systemic conditions: hypertension, diabetes, dyslipidemia, thyroid disease, etc.
- Inflammation marker: neutrophil-to-lymphocyte ratio (NLR).
- Surgical outcomes: intraoperative and postoperative complications.

#### Follow-up

All patients were examined on postoperative day 1 and day 7.

### Statistical analysis

All statistical analyses were performed using R software version 4.2 (R Foundation for Statistical Computing, Vienna, Austria). Eye-level outcomes were analyzed using linear mixed-effects models to account for within-subject correlation when both eyes from the same patient were included. Each model included a random intercept for patient ID, with fixed effects for PEX status (yes/no), age (years), sex (female vs. male), and eye side (OS vs. OD).

No primary outcome was pre-specified in the original clinical audit. *A posteriori*, ACD was chosen as the primary biometric parameter on the basis of previous literature showing it is the most consistently altered measure in PEX eyes [12–14]. A linear mixed-effects model was fitted with ACD as dependent variable, PEX status, age, gender and eye side as fixed effects, and patient identifier as random intercept to account for within-patient correlation between fellow eyes. Secondary outcomes were AL, AQD, LT, CCT and ECD, analysed with the same structure. Model assumptions (residual normality and homoscedasticity) were verified graphically; results are reported as β coefficients with 95% profile-likelihood confidence intervals and two-tailed p-values. Models were estimated by restricted maximum likelihood (REML) using the *lme4* and *lmerTest* packages. Adjusted marginal means and pairwise contrasts were computed using the *emmeans* package. When multiple acquisitions per eye were available, the measurement with the lowest standard deviation or, alternatively, the most recent measurement was selected. Model diagnostics (residual vs. fitted and Q–Q plots) showed no violations of linearity or normality assumptions; continuous variables are expressed as mean ± standard deviation (SD), and categorical variables as counts and percentages. Between-group comparisons were performed using the Welch t-test or Mann–Whitney U test as appropriate, and associations between continuous variables were evaluated with Pearson or Spearman correlation coefficients. Two-sided p-values < 0.05 were considered statistically significant.

To evaluate whether systemic inflammation was associated with biometric parameters or surgical outcomes, the neutrophil-to-lymphocyte ratio (NLR) was analyzed separately. Correlation between NLR and biometric features (AL, ACD, AQD, LT, CCT, ECD) was assessed using Pearson’s or Spearman’s correlation coefficients, depending on variable distribution.

Analyses of surgical complications were restricted to operated eyes only. Because NLR was recorded at the patient level, the same value was assigned to the patient’s operated eye(s).

PEX patients were subdivided into two groups according to surgical outcome — Group 1 (patients with intraoperative or postoperative complications) and Group 2 (uneventful surgery).

Differences in mean NLR values between the two groups were tested using the Mann–Whitney U test, given the non-normal distribution of NLR.

Results were expressed as mean ± SD, with a significance threshold of *p* < 0.05.

The study was conducted in accordance with the Declaration of Helsinki. Patient anonymity was preserved, and aggregated anonymous data was used exclusively for research purposes.

## Results

### Systematic literature review

#### Study selection

A total of 106 records were identified across all databases.

After removing duplicates and applying inclusion/exclusion criteria, 34 studies were included in the qualitative synthesis.

**Fig. 1:**
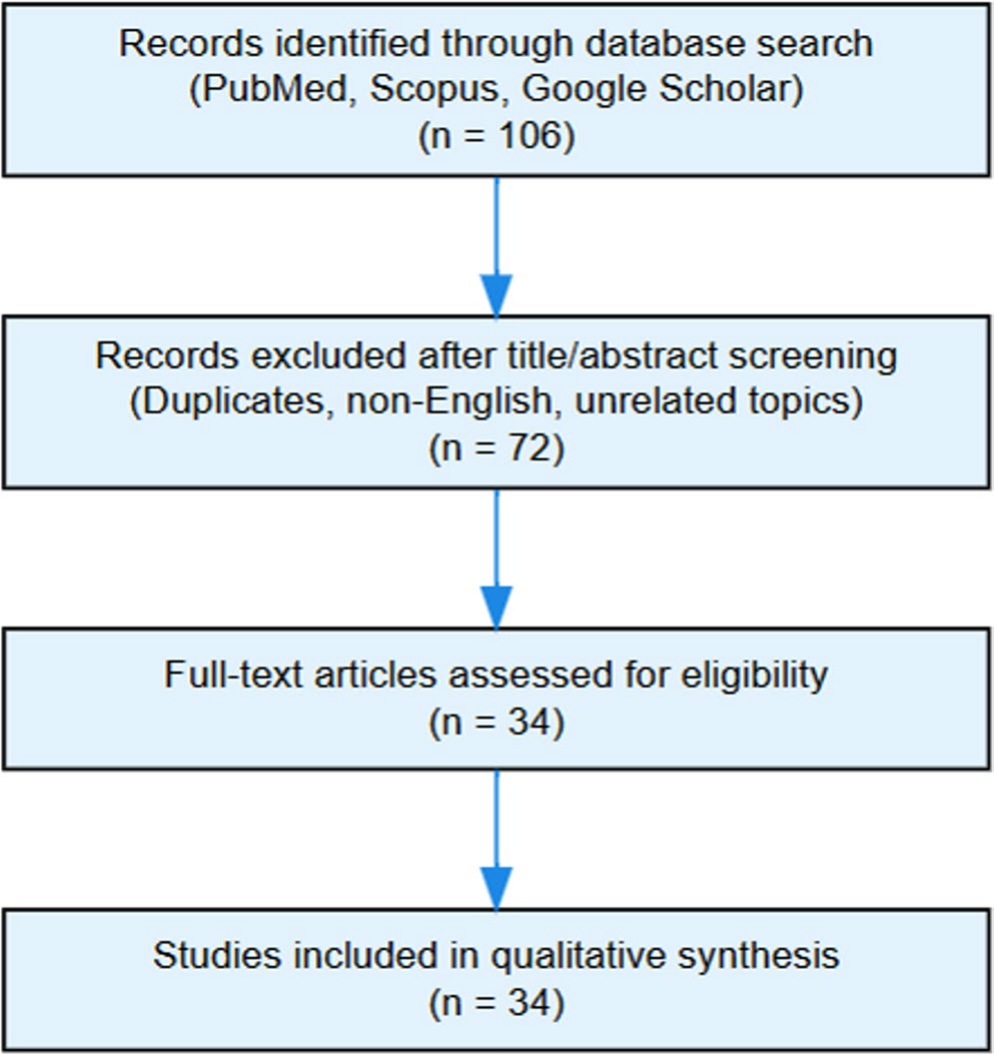
PRISMA 2020 flow diagram of the literature review process. Flowchart summarizing the identification, screening, and inclusion of studies in the systematic literature review on PEX syndrome prevalence. A total of 106 records were identified, 72 were excluded after screening, and 34 studies were included in the qualitative synthesis

A PRISMA 2020 flow diagram summarizing identification, screening, and eligibility steps is shown in Fig. 1.

#### Characteristics of included studies

The included studies encompassed a broad range of populations, including:

- cataract surgery patients.
- general adult populations.
- clinic-based samples.
- screening or epidemiological cohorts.

#### Prevalence of PEX across studies

PEX prevalence showed marked geographic variability, ranging from 0.12% in a large Korean population-based study up to 39.3% in a hospital-based Ethiopian cohort. Mediterranean countries showed wide heterogeneity, with reported prevalence ranging between ~ 3% and 28% in different centers.

#### Demographic trends

Across the included studies, age was consistently associated with higher PEX prevalence, sex distribution showed no consistent pattern; several studies reported associations with elevated intraocular pressure, cardiovascular comorbidities, systemic inflammation, UV exposure, shorter axial length and shallower anterior chamber depth.

A complete summary of prevalence, demographics, population type, and associated factors is provided in Table 1.

**Table 1:** Literature review. NA: not available, SD: standard deviation, IOP: intraocular pressure, AMD: Age-related macular degeneration

Authors	Year	Patients included	Mean age of PEX Patients (years)	Gender ratio (M%)	PEX prevalence	Associated factors	Risk factors	Region	Type of population
Teshome et al. [15]	2004	229	63.7 (SD 10.7)	38.8%M	39.3%	Cataract	High IOP	Ethiopia	Single center patients scheduled for cataract surgery
Andrikopoulos et al. [3]	2009	2140	71.4 (SD 7.5)	NA	27.9% (62.2% bilateral)			Greece	Single center patients scheduled for cataract surgery
Susić N et al. [16]	2006	150	71 unilateral PEX 74.3 bilateral PEX	NA	26%	High IOP, smaller dilated pupillary diameter		Hospital in Croatia	Single center patients scheduled for cataract surgery
Patrice M Hicks et al. [17]	2021	126	74.3	46%M	24,60%			Guatemala	Patients enrolled in an outreach eye camp
Govetto et al.[18]	2015	1763	80.62 (SD 6.3)	45%M	21,2%	High IOP	Age	Spain	Single center patients scheduled for cataract surgery
Kozobolis, et al. [19]	1997	777	NA	21.3%M	16.1%			Crete	Predetermined number of people born and living in various villages, in collaboration with the local birth register offices
Hamad Esehiyb et al. [20]	2021	398	68.7 (SD 7.45)	F: M = 1:1.1	56/398 (14,1%)			East Libya Hospital	Single center patients scheduled for cataract surgery
Rouhiainen et al [21]	1992	833	NA	NA	8.5% in 65-years old, 13.2% in 75-years old			Kuopio (Finland)	Two age groups living in the city of Kuopio, those born in 1914 and 1924
Pedro Romero-Aroca et al. [22]	2011	2342	68.31 (SD 9.60)	50,9%M	13,19% (70,87% unilateral)			Spain	Inhabitants chosen randomly from the population census (age ≥ 60 years old)
Tongabay Cumurcu et al. [23]	2010	831	74.46 (SD 9.18)	52,5%M	12,2% (bilateral 74,2%)	Lower IOP, AMD		Turkey	Single center patients scheduled for cataract surgery
Anastasopoulos et al. [24]	2011	2261	73.8	50%M	11.9% (5.5% bilateral)	Higher IOP, vertical cup disc asymmetry, optic disk damage in either eye		Greece	Cross-sectional, population-based epidemiologic study of chronic eye diseases in the Greek population of Thessaloniki
Gunes et al. [25]	2017	352	74.4 (SD 7.2)	43.5%M	11%	High IOP, higher prevalence of AMD and Glaucoma		Turkey	Single center patients scheduled for cataract surgery
Hugh R. Taylor [5]	1980	82 aborigines	NA	6 M:3 F	9/82 (10.98%)			2 communities of western Australia	50% of the Aborigines age > 30 years old in two settlements in Northwestern Australia
Arnarsson et al. [26]	2007	1045	72 unilateral PEX 71 bilateral PEX	NA	10.7% (bilateral 35/97 cases)		High mean IOP, ACD, LT	Reykjavik	Random sample from the national population census for citizens of Reykjavik, aged ≥ 50 years old
Divija et al. [27]	2024	3649	66.2 (SD 11.67)	67.08%M	10.1% (32.2% bilateral)	Arterial hypertension, diabetes, asthma, coronary artery disease, thyroid disorders, high IOP		Kolar (India)	Single center patients scheduled for cataract surgery
Ugne Rumelaitiene et al. [28]	2020	1033	73.01 (SD 7.97) in PEX group	85 M: 131 F (39,4%)	9.8% study start-34,2% study end		No statistical significance found with investigated risk factors	Lithuania	Random sample of participants from Kaunas city population
Muawyah D. Al-Bdour [19]	2008	1195	68.3 (SD 9.57)	8.4% of women and 9.8% of men included	9,10%	Cataract, glaucoma, phacodonesis		Jordan	Single center patients
Asif Amin Vakil et al. [30]	2012	3000		F: M = 1:1.1	7.10% (84.5% bilateral)			South Kashmir	Hospital-based study, all consenting patients, ≥ 45 years old
Eloy Viso et al. [31]	2010	619	73 (SD 13)	26 M:29 F (47,3%M)	6,50%	Glaucoma, cataract surgery, staining with bengala rose, diabetes	Age > 80	Spain	Age-stratified random sample of 1155 subjects drawn from the population ≥ 40 years old of O Salnés; Of 937 eligible subjects, 619 (66.1%) participate
You QiSheng et al.[32]	2013	3022	63.7 M, 58.1 F	43.2%M	5.82% (45.5% bilateral)	Shorter axial length, shorter anterior chamber depth	Age	China	Population-based, cross-sectional study in Northern China, carried out in 5 communities in the urban district of Haidian in the north of Central Beijing and in 3 communities in the village area of Yufa of the Daxing District south of Beijing
Yildirim et al. [33]	2017	2009	69.1 (SD 9.9)	47%M	5% (38% bilateral)			Turkey	2356 subjects randomly chosen for the sample population based on the database of the Turkish Statistical Agency in Eskisehir; 2017 subjects participated, out of which 2009 were eligible for the study
Arvind et al [34].	2003	2850	64.7 (SD 9.63)	45.4%M	3.8% (50.9% bilateral)			South India	Consecutive subjects ≥ 40 years old from a population based survey in a rural area of southern India
Lingam Vijaya [35]	2016	9600	NA	44,5%M	3,73% (bilateral 40.7%)	Glaucoma, ocular hypertension, narrow angle, age		India	Population-based study conducted to estimate the prevalence of glaucoma in the rural and the urban south Indian populations
Alireza Hashemi et al. [36]	2024	3274	NA	NA	3,63% (81,1% bilateral)		Age, cataract	Iran	Population-based cross-sectional study on individuals ≥ 60 years old in Tehran
Pavičić-Astaloš et al. [37]	2016	5349	NA	33%M	3.6%			North-west Croatia	Hospital based prospective study; age ≥ 40 years old; all patients presenting to the Ophthalmology Polyclinic for various ocular problems
Sulaiman A. Al-Saleh et al. [38]	2015	1967	NA	35 M : 34 F (50,7%),	3,50% (37.7% bilateral)	High IOP, cataract, visual decrease, Flakes on iris margin or trabecular meshwork		Saudi Arabia	Single center patients
A M Abdul-Rahman et al. [39]	2008	2076	68 (SD 11.4)	1240 F	3,40% (bilateral in 24 subjects)	Age, high IOP		Burma	Cross-sectional, population-based survey of the inhabitants ≥ 40 years old in the Meiktila District
Miyazaki et al. [40]	2005	1464	71 (SD 7)	31 F, 19 M	3.4%	Arterial Hypertension	Age	Japan	Cross-sectional population-based survey conducted among residents of Hisayama
Heriot W.J et al. [41]	1984	986	NA	All males	3/986 (3.04%)			Raratonga	Survey of 986 Polynesians in Rarotonga for the complications of diabetes
Kaimbo Wa Kaimbo [42]	2012	2142	70.40 (SD 8)	22 F, 15 M	1.73% (59.46% bilateral)			Congo	Patients aged ≥ 50 who attended the general practice of ophthalmology
Jost B Jonas et al. [43]	2013	4646	NA	46,5%M	1,49% (26% bilateral)		Age	India	Population-based cross-sectional study in Central India; study performed in 8 villages in Kalmeshwar Tehsil, age ≥ 30 years
Arakaki et al [44].	2020	2214	75.9 (SD 8.8)	30 F,14 M	1.3%	Older age, working outdoors		Japan	Population-based survey of all residents aged ≥ 40 years in Kumejima, Okinawa
Ravi Thomas et al [45]	2005	10,293	64.9 (SD 9.8)	47,2%M	0,71% (53.4% bilateral)	Cataract	Age	India	Population-based, cross-sectional epidemiologic study in Andhra Pradesh; 10,293 subjects of all ages from one urban and three rural areas representative of the population
Soa Kim et al [46]	2016	13,817	67.5 (SD 7.3)	5 M:11 F (31,3%)	0,12% (16 patients affected, 6 bilateral)		Age, sun exposure > 5 h daily, cataract	South Korea	Population-based, cross-sectional survey; random sampling of households across 576 national districts, selected to represent the South Korean population

### Cohort study results

#### Study population

During the study period, 3,736 cataract patients were evaluated, contributing 7,237 eyes with usable biometric measurements.

Among these, 99 patients (2.65%) were diagnosed with PEX, with a yearly prevalence of 2.84% in 2024 and 2.44% in 2025.

#### Demographics

PEX patients were significantly older than non-PEX patients (mean age 76.4 ± 6.0 vs. 72.8 ± 8.8 years; Welch *t*-test, *p* < 0.001). Table 2 shows a comparison of age between the two groups.

**Table 2:** Comparison of age distribution between all patients and PEX patients. SD: standard deviation. 1: Welch t-test

	Non-PEX patients	PEX patients	*p*
***Age (years)***	72,8 (SD 8,8)	76,4 (SD 6,0)	**< 0**,**001**^**1**^

Sex distribution was balanced (M: F ratio = 0.98), consistent with literature reports.

#### Comorbidities

The most frequent systemic conditions in PEX patients (Table 3) were hypertension (37.4%), dyslipidemia (25.3%), and diabetes (21.2%). These patterns align with reports linking PEX to systemic vascular and metabolic alterations.

**Table 3:** Features of PEX patients

Monocular/Binocular PEX	45/54
***Associated comorbidities***	***n. (% of the included population)***
***Arterial Hypertension***	37 (37,4%)
***Diabetes***	21 (21,2%)
***Dyslipidemia***	25 (25,3%)
***Dysthyroidism***	7 (7,1%)
***Neoplasia***	8 (8,1%)
***Prostatic Hypertrophy***	14 (14,1%)
***Respiratory***	6 (6,1%)
***Cardiac***	8 (8,1%)

#### Biometric parameters

After adjustment for age, gender and eye side, PEX was not associated with a difference in ACD (β = − 0.07 mm, 95% CI − 0.21 to 0.07, *p* = 0.335), nor with axial length (AL), aqueous depth (AQD), lens thickness (LT), or central corneal thickness (CCT) (all *p* > 0.05). Older age and female sex were independently associated with shallower anterior chambers and thicker lenses (*p* < 0.01 for both), consistent with known biometric aging trends.

Table 4 summarizes the fixed-effect estimates, 95% confidence intervals, and p-values for each model.

**Table 4:** Multivariable mixed-effects analysis of biometric parameters. PEX = pseudoexfoliation syndrome; ACD = anterior chamber depth; AQD = aqueous depth; LT = lens thickness; CCT = central corneal thickness; AL = axial length; CI = confidence interval; OD = right eye; OS = left eye

Parameter	Predictor	Estimate	Std. Error	95% CI (Lower; Upper)	*p*-value
**ACD (mm)**	PEX (yes vs. no)	–0.069	0.072	–0.210; 0.072	0.335
	Age (per year)	–0.0051	0.0011	–0.0072; − 0.0030	**< 0.001**
	Female sex	–0.142	0.020	–0.182; − 0.102	**< 0.001**
	Eye side (OS vs. OD)	–0.067	0.018	–0.102; − 0.032	**< 0.001**
**AQD (mm)**	PEX (yes vs. no)	–0.068	0.072	–0.209; 0.074	0.348
	Age (per year)	–0.0049	0.0011	–0.0070; − 0.0028	**< 0.001**
	Female sex	–0.140	0.020	–0.180; − 0.101	**< 0.001**
	Eye side (OS vs. OD)	–0.068	0.018	–0.103; − 0.033	**< 0.001**
**LT (mm)**	PEX (yes vs. no)	+ 0.212	0.137	–0.056; 0.480	0.121
	Age (per year)	+ 0.0050	0.0021	+ 0.001; +0.009	**0.015**
	Female sex	–0.047	0.039	–0.123; 0.029	0.221
	Eye side (OS vs. OD)	+ 0.165	0.039	+ 0.090; +0.241	**< 0.001**
**CCT (µm)**	PEX (yes vs. no)	–0.003	0.004	–0.011; 0.006	0.562
	Age (per year)	–0.00020	0.00006	–0.00032; − 0.00007	**0.002**
	Female sex	–0.0020	0.0012	–0.0044; +0.0005	0.116
	Eye side (OS vs. OD)	+ 0.0005	0.0004	–0.0003; +0.0014	0.211
**AL (mm)**	PEX (yes vs. no)	–0.189	0.170	–0.523; +0.145	0.267
	Age (per year)	–0.039	0.0025	–0.044; − 0.034	**< 0.001**
	Female sex	–0.536	0.048	–0.629; − 0.443	**< 0.001**
	Eye side (OS vs. OD)	–0.024	0.015	–0.052; +0.005	0.108

#### Inflammatory marker (NLR) and ocular parameters

**Fig. 2:**
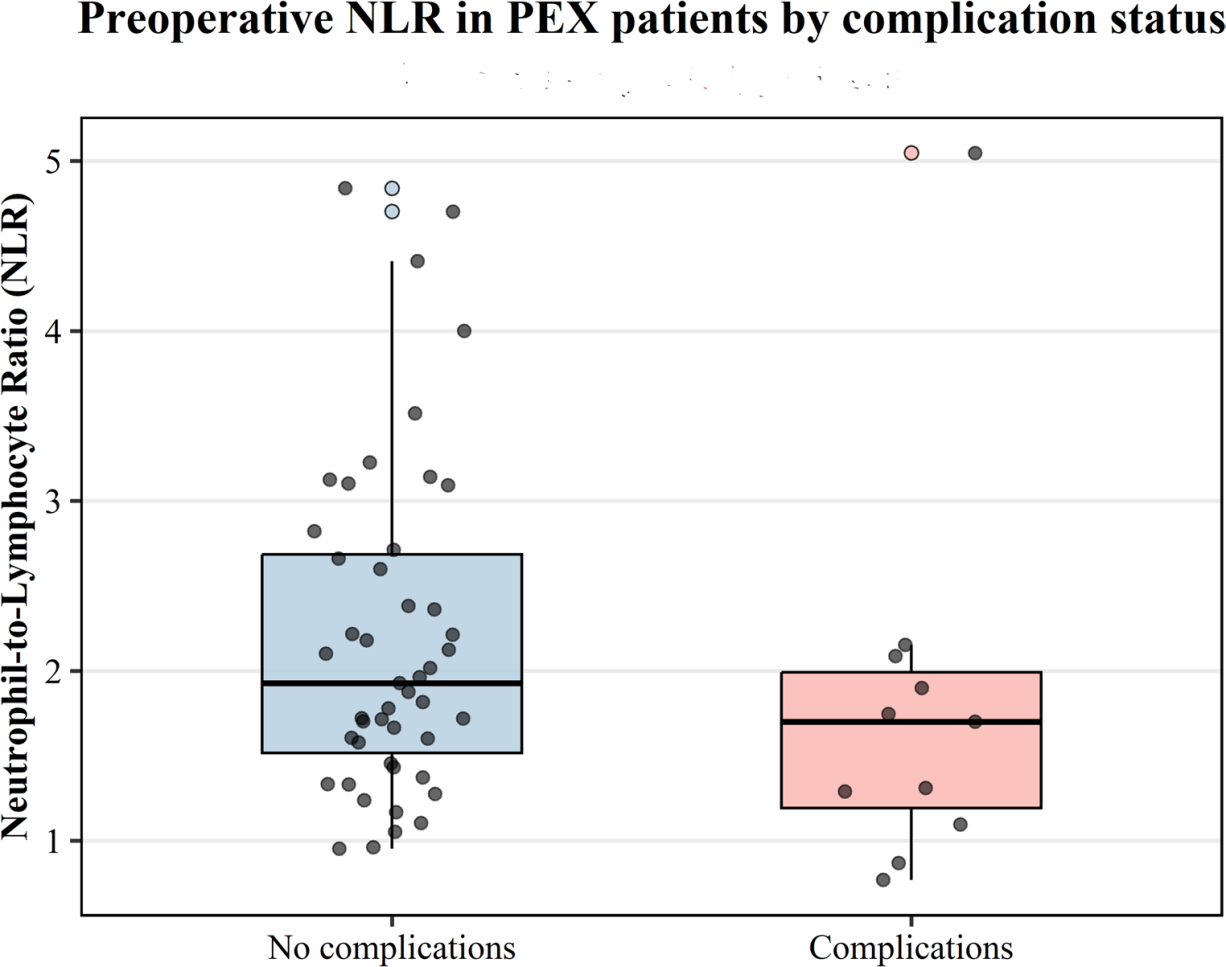
Boxplot showing preoperative neutrophil-to-lymphocyte ratio (NLR) values among PEX patients who underwent cataract surgery, stratified by the presence or absence of intraoperative or postoperative complications

No significant correlation was observed between NLR and biometric parameters. Among PEX patients with available NLR, values did not differ significantly between those with intra-/postoperative complications and those without (median [IQR] 1.72 [0.74] vs. 1.94 [1.18], Mann–Whitney U, *p* = 0.081; *n* = 14 vs. 60) (Fig. 2). These findings indicate that, although age and sex influence ocular biometry, systemic inflammatory status as reflected by NLR does not appear to modulate biometric profiles or surgical risk among PEX patients (Table 5).

**Table 5:** Comparison of mean NLR value for group 1 and group 2; ^1^: Mann-Whitney U test

	Group 1	Group 2	*p*^1^
***Mean NLR value***	1,82	2,20	0.081

#### Summary of PEX biometric features

The main biometric features of PEX patients are summarized in Table 6.

**Table 6:** Biometric features, corneal features and NLR of patients affected by PEX. AC: anterior chamber Depth, LT: lens Thickness, AL: axial Length, ECD: endothelial cell Density, CCT: central corneal Thickness, AQD: aqueous depth, NLR: Neutrophil/Lymphocyte ratio

	ACD (mm)	LT (mm)	AL (mm)	ECD (cell/mm^2^)	CCT (µm)	AQD (mm)	NLR
***Mean***	*3*,*73*	*4*,*31*	*23*,*01*	*2296*,*4*	*510*,*5*	*2*,*82*	*2*,*121*
***Standard deviation***	*0*,*4594*	*1*,*390*	*2*,*269*	*479*,*04*	*60*,*54*	*0*,*78*	*1*,*015*

Values were broadly within expected ranges and aligned with normative data reported in previous studies.

## Discussion

This study provides a combined perspective on PEX by integrating a systematic review of published prevalence data with a detailed hospital-based cohort analysis from a Southern Italian population. This dual approach allows to contextualize our findings within global epidemiological trends while highlighting local demographic and clinical characteristics of PEX patients undergoing cataract surgery.

### Interpretation of prevalence findings in context of the literature

**Fig. 3:**
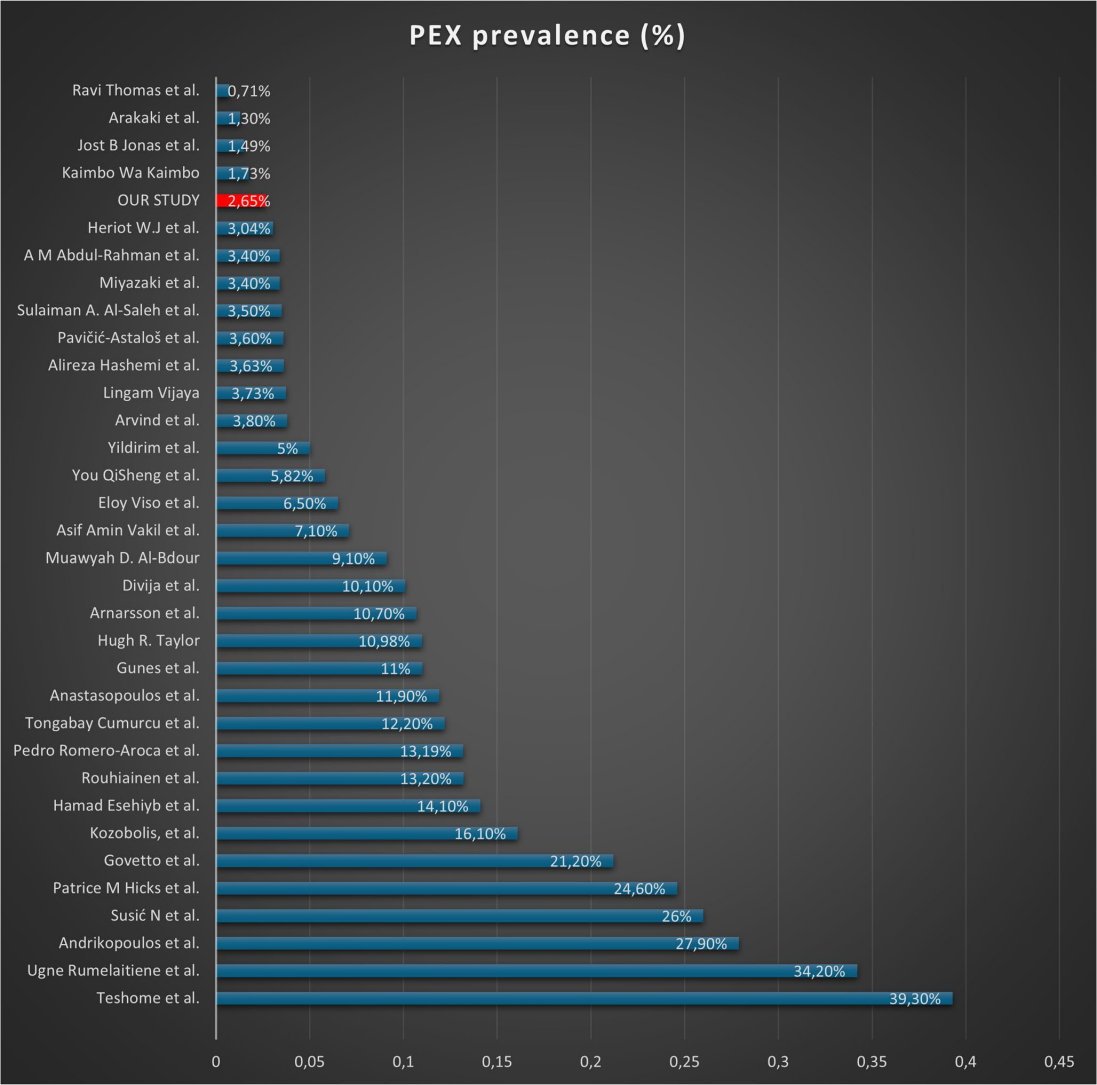
PEX prevalence in our literature review

The prevalence of PEX in our cohort (2.65%) was relatively low compared with many Mediterranean reports, where rates often exceed 10% (Fig. 3). The systematic review showed substantial geographic heterogeneity, with values ranging from < 1% in East Asian population-based cohorts [46] to > 30% in hospital-based studies from Ethiopia [15] and parts of the Mediterranean region [37]. It is also possible to see high differences in the Mediterranean region, with values that span from about 28% in Greece [3] to 3.4% in northwest Croatia [37]. Remarkably, in the same Croatia, (Fig. 3) another study reports a prevalence of 26% [16].

Such variability reflects differences in genetics, UV exposure, ethnic composition, diagnostic protocols, and study design. Our prevalence estimate (2,65%) derives from a single-center, hospital-based cohort, which inherently limits external validity. Such samples are influenced by care-seeking patterns, referral bias, and the demographic profile of the catchment area, and therefore should not be interpreted as population-based prevalence. Conversely, population-based studies - though more representative - often lack the precision needed to detect relatively uncommon entities such as PEX.

To enhance interpretability, we contextualized our estimate within both clinic- and population-based reports (Table 1), highlighting how study design substantially affects prevalence variability. Beyond environmental and methodological heterogeneity, genetic susceptibility plays a pivotal role in PEX pathogenesis. Variants in the LOXL1 gene and related loci (e.g., CNTNAP2, CLCN7) [47–50] have been strongly associated with disease expression across populations, potentially accounting for regional and ethnic differences in prevalence and phenotype. While our study was not designed to explore genotype–phenotype correlations, this genetic framework provides a biological rationale for the geographic variability observed in our review.

### Demographic characteristics and systemic associations

**Fig. 4:**
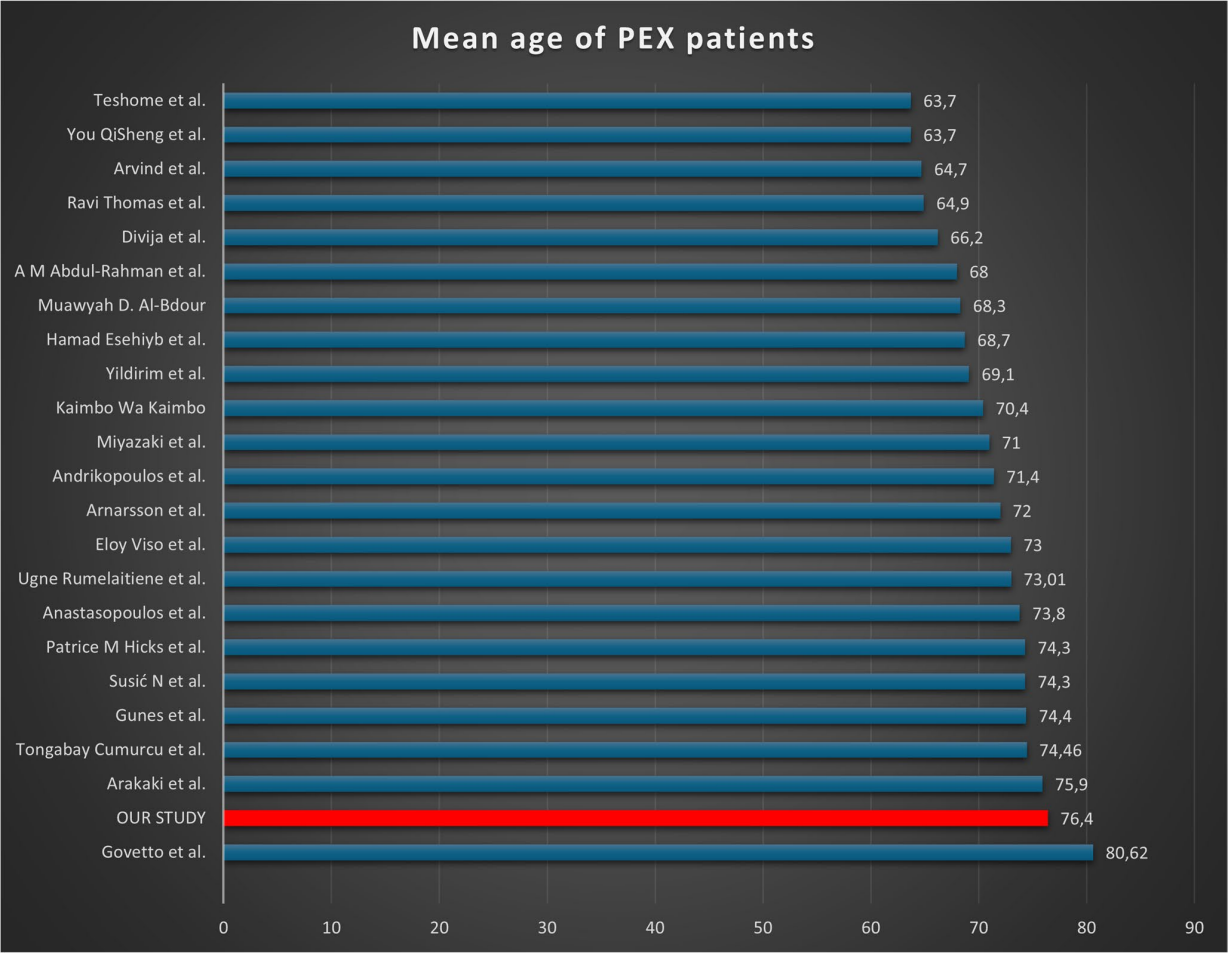
PEX patients’ mean age in the studies in our literature review

The mean age in our cohort of PEX patients was among the highest reported in the literature, confirming age as one of the strongest risk factors for PEX development. This likely reflects both the older age profile of the Italian population and the demographic characteristics of patients referred to our surgical unit. As observed in previous studies, (Fig. 4) the prevalence of PEX increases steadily with advancing age, supporting its degenerative and cumulative nature.

**Fig. 5:**
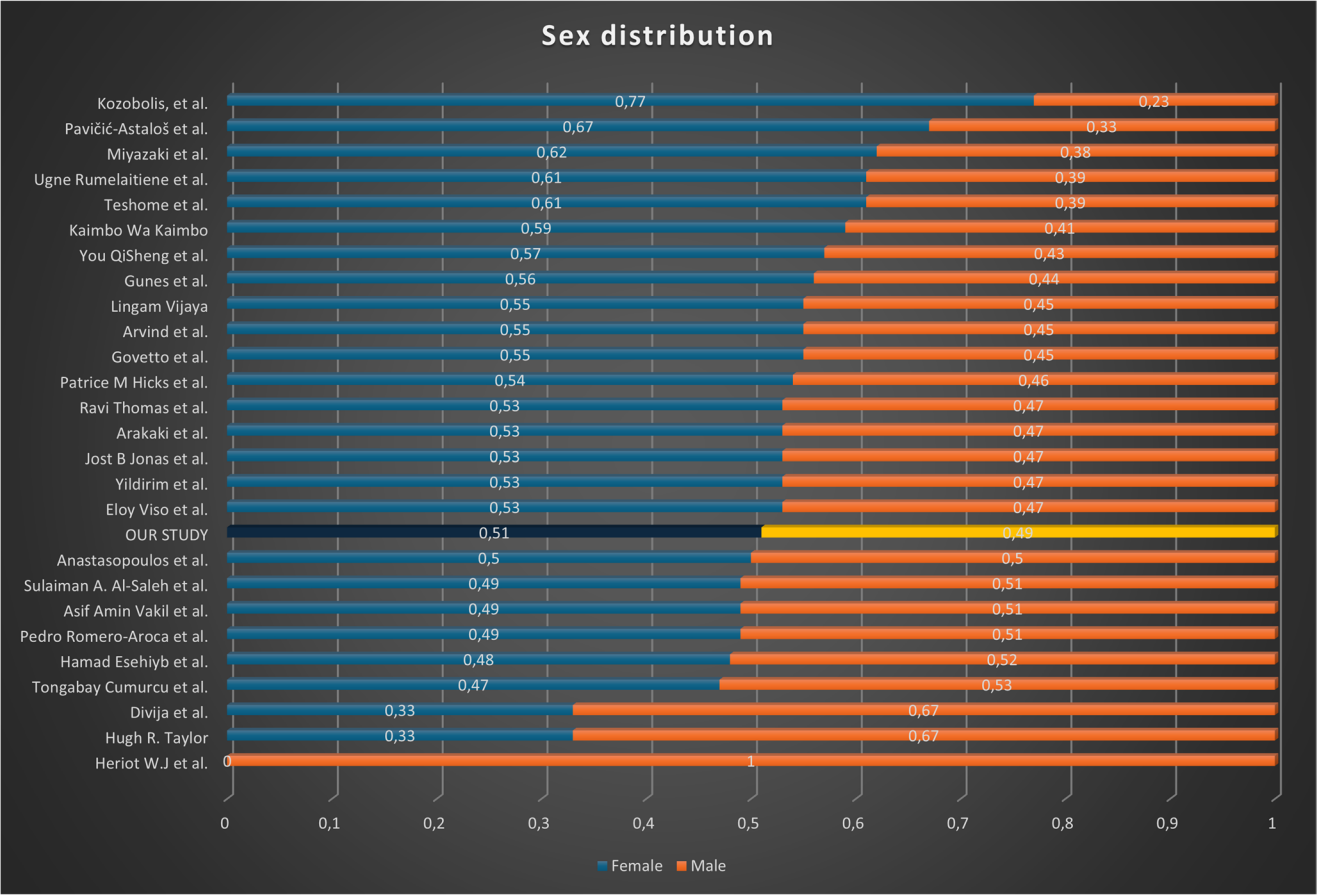
Sex distribution in the literature review

In our cohort, the distribution of PEX by sex was balanced (M: F ratio = 0.98), showing no significant difference between males and females. This finding is consistent with most of the literature (Fig. 5), which generally reports either no sex predominance or a slight, non-significant female excess. Minor discrepancies between studies are likely related to differences in sample size, population structure, and regional demographics.

Systemic comorbidities were common among PEX patients in our cohort. Arterial hypertension was the most prevalent condition (37.4%), followed by dyslipidemia (25.3%) and diabetes mellitus (21.2%). These findings confirm the results of a previous study, suggesting a potential vascular component in PEX pathogenesis, as hypertension and dyslipidemia have been implicated in extracellular matrix abnormalities and elastosis, which are key features of PEX [51]. In a recent review, the association between PEX and cardiovascular system diseases was assessed, finding a relationship between this syndrome and hypertension, angina or a history that combined angina, myocardial infarction or stroke [52]. Furthermore, a link between PEX and coronary artery disease was found in a study that included 50 patients with coronary artery disease assessed by coronary angiography and 50 controls [53]. A 2017 research found that patients affected by PEX showed a higher level of blood low-density lipoprotein (LDL) when compared to a control group, also finding an association with diabetes mellitus and arterial hypertension [54]. These findings could be explained if we consider that PEX affects mainly older people, but we should also consider that PEX pathogenesis includes an inflammatory component.

### Ocular biometry in PEX: alignment with prior evidence

From a clinical perspective, after accounting for within-patient correlation and adjusting for age, sex, and eye side, PEX was not independently associated with major biometric parameters such as AL, ACD, LT, AQD, or CCT.

We acknowledge that the choice of ACD as *post-hoc* primary outcome reflects literature-based plausibility rather than pre-planned hypothesis testing; nevertheless, it provides a clear hierarchical testing framework. The mixed-effects approach appropriately accounts for correlation between fellow eyes, yielding unbiased standard errors.

This observation suggests that the surgical complexity often encountered in PEX—such as zonular instability, inadequate dilation, and increased risk of posterior capsule rupture—is driven more by tissue fragility and extracellular matrix abnormalities than by variations in standard biometric measurements. Consequently, biometry alone may not adequately predict operative difficulty in PEX patients.

### Systemic inflammation (NLR) and surgical outcomes

Consistent with this interpretation, NLR showed no significant relationship with either biometric parameters or postoperative complications, reinforcing the notion that systemic inflammation may not directly influence ocular anatomic risk in these patients.

Finally, the limited number of PEX patients with complete biometry and NLR data (*n* = 99) constrains statistical power, particularly for rare outcomes such as intra- or postoperative complications, increasing the likelihood of Type II errors. Unmeasured confounders—including smoking status, sunlight exposure, or genetic background—were not available and could contribute to residual bias. These limitations are acknowledged in the interpretation of our results and underscore the need for larger, multicenter studies with integrated clinical and genetic profiling.

A study that involved a central Indian population to examine PEX data showed also no relationship between PEX presence and biometric values [43]. The average values of the included parameters are substantially aligned with the normal ranges [55–58].

When PEX patients were stratified according to surgical outcomes, mean NLR values did not differ significantly between those who experienced intraoperative or postoperative complications and those with uneventful surgery. Although previous reports have suggested that higher NLR may predict perioperative complications in PEX eyes [10], our findings do not support this association.

Differences may be explained by the small number of complication events, variability in inflammatory status over time, or population-related factors. Overall, NLR cannot be considered a reliable predictor of surgical risk in this cohort.

### Strengths and limitations

This study provides novel data on the epidemiology and clinical profile of PEX in a subregion of Apulia, offering insights from an area not previously reported in the literature. However, several methodological limitations must be considered.

First, the single-center, hospital-based design may introduce selection bias and limit the generalizability of our findings, as the sample likely reflects the demographic and clinical profile of patients referred for cataract surgery rather than the general population.

Second, although the number of PEX cases was consistent with the relatively low prevalence reported in Southern Europe, the sample size was limited for detecting subtle associations between PEX, biometric parameters, or postoperative complications.

Third, the inclusion of both eyes in the biometry analysis may introduce selection imbalance if the non-operated fellow eye differs systematically from the operated one (e.g., earlier disease stage or anisometropia). This potential bias was mitigated by modeling at the eye level with a patient-level random intercept and by restricting complication analyses to operated eyes only.

Fourth, the retrospective nature of the analysis prevented adjustment for potentially relevant confounders such as ultraviolet exposure, smoking habits, or systemic inflammatory and metabolic variables beyond NLR.

Finally, because of the cross-sectional design, causal inferences cannot be drawn.

Future research should aim to include larger, multicenter, and prospective cohorts, ideally integrating genetic data (e.g., *LOXL1* polymorphisms) and systemic inflammatory markers. Such studies could help clarify the interplay between ocular biometry, systemic vascular health, and PEX pathogenesis, improving risk stratification and perioperative management.

## Conclusions

PEX syndrome was identified in 2.65% of cataract patients operated at our center, representing a relatively low prevalence compared with other Mediterranean cohorts. PEX patients were significantly older than non-PEX subjects, confirming age as the main risk factor for the condition. The sex distribution was balanced, consistent with most previous studies.

Arterial hypertension was the most frequent systemic comorbidity, supporting the hypothesis of a vascular component in PEX pathogenesis. However, no significant associations were found between PEX and biometric parameters or between the NLR and either biometry or postoperative complications.

Overall, our findings suggest that while PEX remains primarily an age-related degenerative condition, systemic inflammatory status as captured by NLR does not appear to influence ocular biometry or surgical risk. Larger, multicenter, and prospective studies integrating genetic and systemic inflammatory markers are warranted to further elucidate the complex interplay between ocular and systemic factors in PEX.
